# *Saccharomyces cerevisiae ASN1* and *ASN2* are asparagine synthetase paralogs that have diverged in their ability to polymerize in response to nutrient stress

**DOI:** 10.1038/s41598-018-36719-z

**Published:** 2019-01-22

**Authors:** Chalongrat Noree, Naraporn Sirinonthanawech, James E. Wilhelm

**Affiliations:** 1 Institute of Molecular Biosciences, Mahidol University, 25/25 Phuttamonthon 4 Road, Salaya, Phuttamonthon, Nakhon Pathom 73170 Thailand; 2 Section of Cell and Developmental Biology, Division of Biological Sciences, University of California, San Diego, 9500 Gilman Drive (MC 0347), La Jolla, CA 92093-0347 USA

## Abstract

Recent work has found that many metabolic enzymes have the ability to polymerize in response to metabolic changes or environmental stress. This ability to polymerize is well conserved for the few metabolic enzyme paralogs that have been studied in yeast. Here we describe the first set of paralogs, Asn1p and Asn2p, that have differential assembly behavior. Asn1p and Asn2p both co-assemble into filaments in response to nutrient limitation. However, the ability of Asn2p to form filaments is strictly dependent on the presence of Asn1p. Using mutations that block enzyme activity but have differential effects on Asn1p polymerization, we have found that Asn1p polymers are unlikely to have acquired a moonlighting function. Together these results provide a novel system for understanding the regulation and evolution of metabolic enzyme polymerization.

## Introduction

Genome duplication is a source of both evolutionary novelty and genetic robustness. However, these two possibilities are in tension with each other since paralogs diverge or acquire secondary functions, the ability of one paralog to compensate for the other is decreased. In *Saccharomyces cerevisiae*, approximately 30% of the genes are duplicated^1^, making yeast an excellent system for understanding how paralogs might acquire moonlighting functions.

Recent works on yeast metabolic enzymes have identified a subset of enzymes that are capable of forming visible intracellular structures in response to nutrient limitation^2–5^. While the assembly of several of these structures is tightly linked to the regulation of enzyme activity, there have been suggestions that some of these filaments may have acquired secondary “moonlighting” functions over evolution. For instance, while CTP synthetase forms filaments that are conserved from *Escherichia coli* to mammals^4,6–8^, in *Caulobacter*, the formation of these filaments affects cell shape suggesting they might have an additional cytoskeletal role^6^. These findings suggested that the identification of paralogs with differential polymerization behavior would be an ideal route to understand both how metabolic enzymes polymerize as well as determine if a given metabolic filament had acquired an evolutionarily novel function.

Here we find that Asn1p and Asn2p, the paralogs of yeast asparagine synthetase, can co-assemble into a common filament. However, only Asn1p is capable of filament formation when its paralog is absent. We have also identified inactivating mutations in *ASN1* that differentially affect Asn1p polymerization. Our analysis of these mutations argues that Asn1p activity is not required for polymerization, but that the synthetase domain of the enzyme is a key contributor to filament assembly. Furthermore, inactivating mutations in Asn1p that differentially affect polymerization have no effect on growth in rich media arguing that Asn1p filaments have not acquired a second, “moonlighting” function. Together these results suggest that the main role of Asn1p polymerization is to regulate enzyme activity and suggest that metabolic enzyme paralogs will be useful tool for understanding the role of enzyme polymerization *in vivo*.

## Results

### Asn1p and Asn2p co-assemble into a common filament

**Figure 1:**
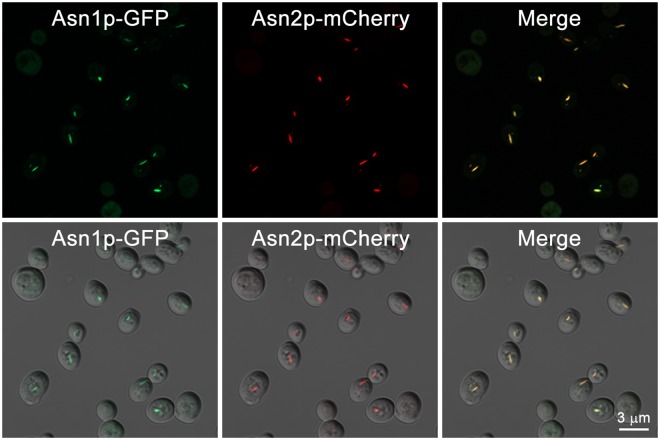
Asn1p and Asn2p colocalize to the same intracellular structures. Yeast *ASN1::GFP ASN2::mCherry* was grown in YPD at 30 °C with shaking for 5 days, fixed with 3.36% formaldehyde before imaging. Cells were captured in Z-stack for approximately 1–3 μm, and projected into 2D image using maximum intensity. Lower panel shows fluorescent images merged with DIC.

The yeast paralogs of asparagine synthetase, Asn1p and Asn2p, were both previously found to form filaments in response to nutrient stress^3,5^. However, it was unclear if Asn1p and Asn2p assembled into a common filament or polymerized into distinct filaments. In order to distinguish between these two possibilities, we performed colocalization experiments using a yeast strain where Asn1p was tagged with GFP and Asn2p was tagged with mCherry. When yeast cells were grown to stationary phase (5 days) to trigger robust asparagine synthetase filament assembly, both Asn1p-GFP and Asn2p-mCherry colocalized to the same filaments (Fig. 1). Thus, Asn1p and Asn2p can co-assemble into a common filament.

### Without Asn2p, Asn1p can still assemble into visible structures, but not *vice versa*

**Figure 2:**
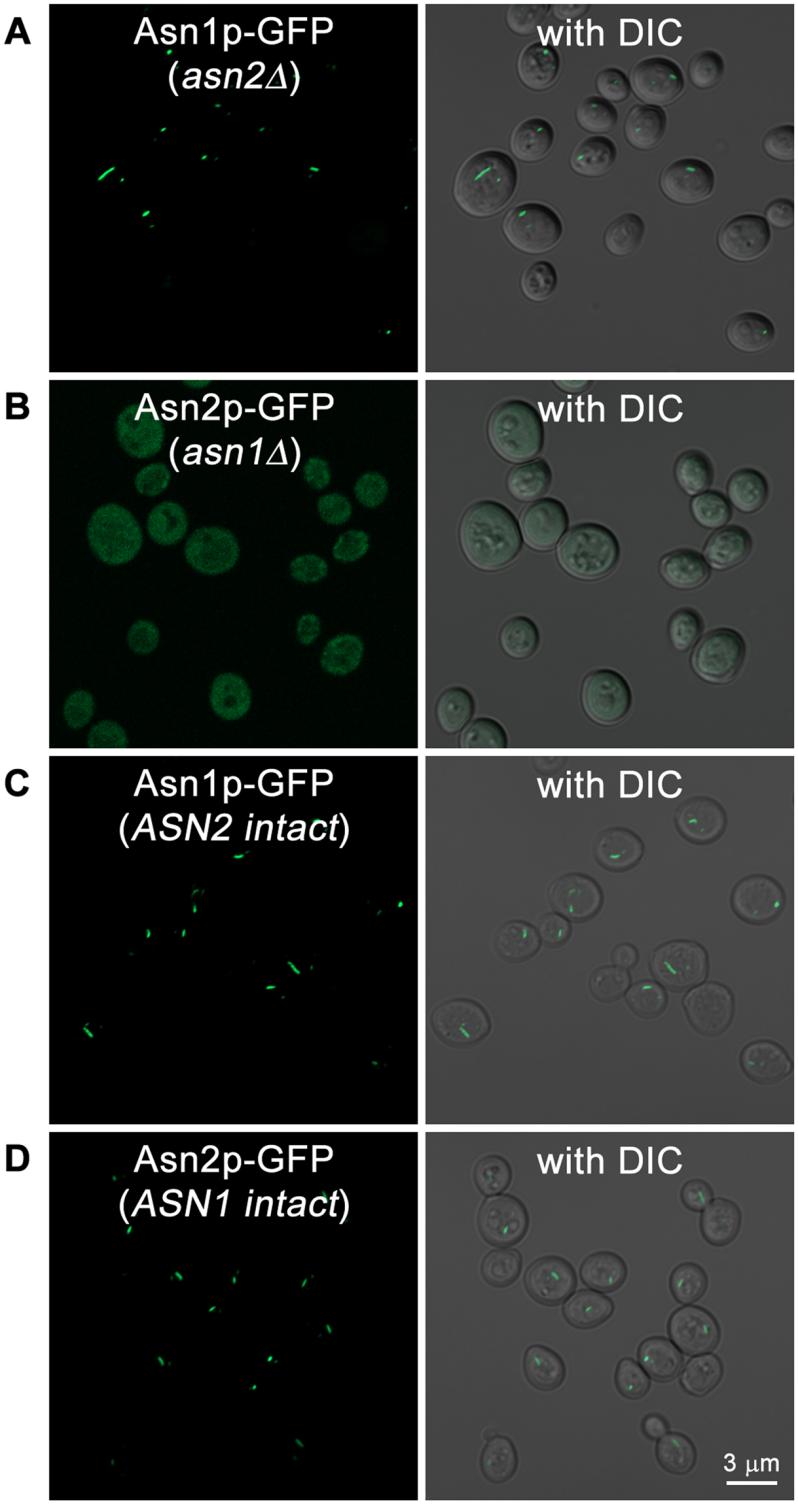
Asn1p can form cytoplasmic structures in the absence of Asn2p, but not vice versa. Yeast strains (**A**) *ASN1::GFP asn2Δ*, (**B**) *ASN2::GFP asn1Δ*, (**C**) *ASN1::GFP* (*ASN2* intact; from the yeast GFP collection), and (**D**) *ASN2::GFP* (*ASN1* intact; from the yeast GFP collection) were grown in YPD at 30 °C with shaking for 5 days, fixed with 3.36% formaldehyde before imaging. Cells were captured in Z-stack for approximately 1–3 μm, and projected into 2D image using maximum intensity. Right panel shows fluorescent images merged with DIC. We observed no difference in the growth rates of *ASN1::GFP asn2Δ* and *ASN2::GFP asn1Δ* during log phase, saturation, or stationary phase.

In order to determine if each asparagine synthetase paralog was equally capable of polymerization, we next tested the ability of Asn1p and Asn2p to form filaments when its paralog was deleted. When *ASN1::GFP asn2Δ* strains were grown to stationary phase, Asn1p formed robust filaments indicating that Asn1p polymerization was not dependent on the presence of Asn2p. In contrast, when *ASN2::GFP asn1Δ* strains were grown to stationary phase, no Asn2p filaments were present (Fig. 2). This argues that the yeast asparagine synthetase paralogs, Asn1p and Asn2p, have diverged in their ability to polymerize, but that Asn2p still retains the ability to incorporate into an Asn1p filament.

As the unequal protein expression levels between Asn1p and Asn2p would be a concern that this might cause the assembly defect of Asn2p in the absence of Asn1p, we performed Western blot analysis of yeast strains *ASN1::GFP* (in *asn2*Δ) and *ASN2::GFP* (in *asn1*Δ). At day 5, the expression levels of Asn1p-GFP (without Asn2p) and Asn2p-GFP (without Asn1p) were comparable (Supplementary Fig. S1). Thus, the Asn2p assembly defect was not because of its expression levels.

### Inactivating distinct steps of the asparagine synthetase reaction has differential effects on polymerization

As a first step to identifying mutations that separate enzyme activity from polymerization, we focused our attention on known mutations that disrupt distinct steps in asparagine synthetase reaction. Asparagine synthetase has two functional domains: a glutamine amidotransferase (or GAT) domain located at N-terminus and synthetase domain at C-terminus. The glutamine amidotransferase is responsible for catalyzing glutamine hydrolysis, yielding glutamate and ammonia (glutamine + H_2_O → glutamate + NH_3_). The NH_3_ is then transferred to the synthetase domain where aspartate is finally converted to asparagine (aspartate + ATP + NH_3_ → asparagine + AMP + PP_i_)^9,10^.

Previous studies identified mutations that disrupt either the first or second step of the asparagine synthetase reaction. C1A and C1Δ mutations disrupt the glutamine amidotransferase function of asparagine synthetase^11–13^, while human R340A or bacterial R325A mutations have been shown to block the transfer of NH_3_ by the synthetase domain of asparagine synthetase^14–16^. Thus, these two mutations (equivalent to C1A and R344A, respectively, in yeast Asn1p; amino acid sequence alignment shown in Supplementary Fig. S2) presented an opportunity to determine if inactivating different steps of the enzyme reaction had similar or different effects on Asn1p filament formation.

**Figure 3:**
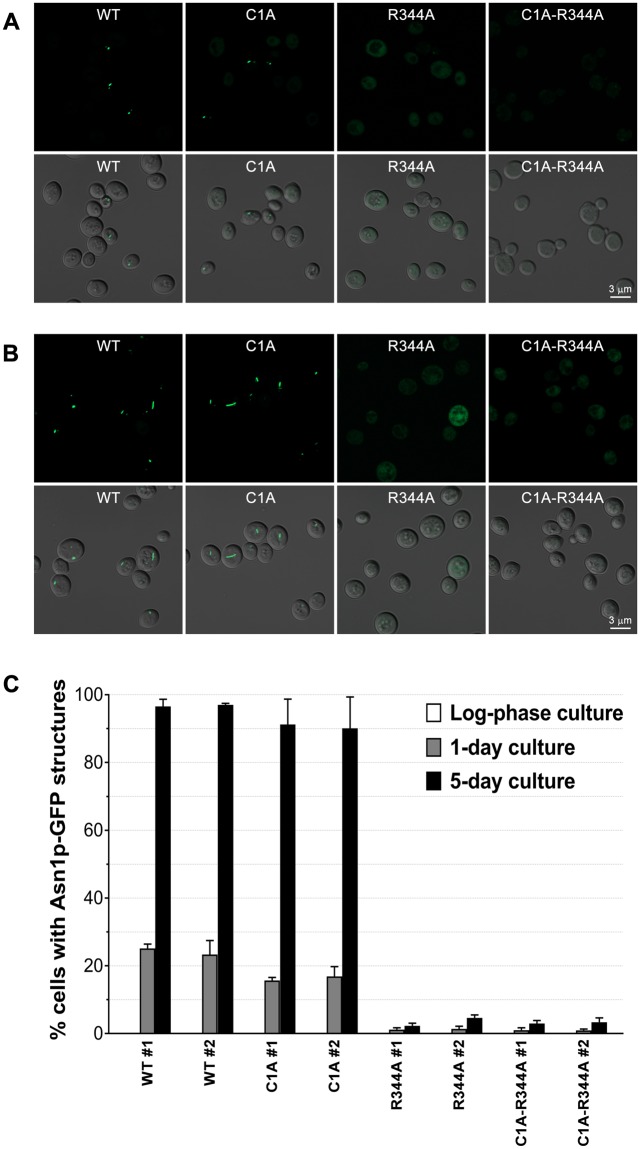
R344A single mutation and C1A-R344A double mutation show Asn1p-GFP assembly defect. Yeast *ASN1(WT)::GFP asn2Δ*, *asn1(C1A)::GFP asn2Δ*, *asn1(R344A)::GFP asn2Δ*, and *asn1(C1A-R344A)::GFP asn2Δ* were grown in YPD at 30 °C with shaking for 1 day (**A**) and 5 days (**B**), fixed with 3.36% formaldehyde before imaging. Cells were captured in Z-stack for approximately 1–3 μm, and projected into 2D image using maximum intensity. Lower panels show fluorescent images merged with DIC. (**C**) Structure formation frequency of Asn1p-GFP at exponential phase, day 1, and day 5, in the yeast strains mentioned above. For each strain, two different clones were used for analysis. Three independent experiments were performed and reported as average ±SEM.

We first tested whether blocking the glutamine amidotransferase function of Asn1p affected polymerization. We constructed yeast strains that expressed C1A Asn1p-GFP as the only form of asparagine synthetase to examine filament formation when the cells were grown to saturation (1 day) or stationary phase (5 days). (Fig. 3, Table 1). We observed no significant defect in filament assembly arguing that N-terminal amidotransferase domain is not associated with Asn1p assembly and that enzyme activity is not required for polymerization. In contrast, when we constructed yeast strains that expressed R344A Asn1p-GFP as the only form of asparagine synthetase we observed little to no filament assembly at either saturation or growth to stationary phase (Fig. 3, Table 1). This result argues that not all inactivating mutations have an equivalent effect of Asn1p polymerization and implicates the C-terminal synthetase domain in Asn1p filament formation. Similarly, Asn1p-GFP bearing both C1A and R344A mutations failed to form filaments (Fig. 3, Table 1). These differences in filament formation are not due to changes in protein levels (Supplementary Fig. S5). Together these results argue that Asn1p polymerization is linked to the function of the synthetase domain and that it is possible to identify inactivating alleles of Asn1p that have differential effects on polymerization.

**Table 1:** Structure formation frequency and length distribution analysis of indicated yeast strains grown under indicated culture conditions.

Yeast strain	Clone #	% cells with Asn1p-GFP structures (average ± SEM)		
		Log-phase	Day 1	Day 5
*ASN1::GFP* (from the yeast GFP collection)	—	0.0 ± 0.00	26.3 ± 2.64	96.4 ± 0.71
*ASN1(WT)::GFP asn2Δ*	1	0.0 ± 0.00	25.1 ± 0.75	96.6 ± 1.22
	2	0.0 ± 0.00	23.3 ± 2.41	97.0 ± 0.29
*asn1(C1A)::GFP asn2Δ*	1	0.0 ± 0.00	15.6 ± 0.55	91.2 ± 4.32
	2	0.0 ± 0.00	16.9 ± 1.68	90.1 ± 5.35
*asn1(R344A)::GFP asn2Δ*	1	0.0 ± 0.00	1.2 ± 0.30	2.3 ± 0.43
	2	0.0 ± 0.00	1.4 ± 0.45	4.7 ± 0.49
*asn1(C1A-R344A)::GFP asn2Δ*	1	0.0 ± 0.00	1.0 ± 0.39	2.9 ± 0.53
	2	0.0 ± 0.00	0.9 ± 0.23	3.4 ± 0.73
P-value (C1A vs. WT)		—	0.1211^ns^ (two-tailed)	0.0810^ns^ (two-tailed)
P-value (R344A vs. WT)		—	0.0279* (two-tailed)	0.0066** (two-tailed)
P-value (C1A-R344A vs. WT)		—	0.0237* (two-tailed)	<0.0001**** (two-tailed)
**Yeast Strain**		**Length of asparagine synthetase structure at day 5 (μm)**		
		**Max**	**Min**	**Average**
*ASN1::GFP* (from the yeast GFP collection)		3.876	0.108	0.787
*ASN2::GFP* (from the yeast GFP collection)		2.682	0.108	0.734
*ASN1(WT)::GFP asn2Δ*		3.086	0.108	0.864
*asn1(C1A)::GFP asn2Δ*		3.820	0.108	1.041

Note: 300 structures were randomly captured and analyzed for each strain. “ns” indicates not significant (p>0.05), whereas the asterisks indicate significant difference (p<0.05) between WT and mutants.

### Asn1p filaments do not have a moonlighting function that affects yeast growth

The divergence in the polymerization behavior of Asn1p and Asn2p suggested that the Asn1p filament might have acquired a second “moonlighting” function. However, a previous screen to identify moonlighting enzymes comparing catalytic inactivating mutations to null mutations did not identify Asn1p^17^. This screen compared the effects of C1A mutation that inactivates Asn1p with null alleles of *ASN1* under 11 growth conditions. Since we have found that the C1A mutation does not affect filament formation, these results argue that the filaments do not have a moonlighting function under these conditions. In order to further test this conclusion, we also compared the effects of the C1A mutation with the R344A mutation that blocks both the polymerization and enzyme activity of Asn1p (Supplementary Fig. S3). We observed no growth difference between wild type *ASN1::GFP asn2Δ*, *asn1(C1A)::GFP asn2Δ*, *and asn1(R344A)::GFP asn2Δ* arguing that Asn1p filaments have no moonlighting function for yeast growing in rich media.

## Discussion

While the number of metabolic enzymes that are capable of forming novel intracellular structures continues to increase, the ability to generate separation of function alleles that can be used to test for either a role for polymerization in regulating enzyme activity or a “moonlighting” role for the enzyme continues to be limiting. One potential way around this bottleneck is to use paralogous pairs of enzymes that have diverged in their ability to polymerize. Here we have identified the first such paralogous pair, Asn1p and Asn2p, that have distinct abilities to polymerize. Even though Asn1p and Asn2p are 88% identical at the amino acid level (Supplementary Fig. S4), Asn1p can form filaments in the absence of Asn2p while Asn2p requires Asn1p for incorporation into a filament. This differential behavior is distinct from that of the best characterized set of paralogous polymerizing enzymes in yeast, the CTP synthetases, encoded by *URA7* and *URA8*. Since both Ura7p and Ura8p are capable of polymerization and are seemingly interchangeable it was largely thought that polymerization would be an essential feature that would be maintained between functional paralogs^18^. The fact that Asn1p and Asn2p have diverged in their polymerization behavior suggests that different selective pressures may be acting on asparagine synthetase paralogs relative to CTP synthetase paralogs. We tested one potential source for this difference: the possibility that Asn1p filaments acquired a moonlighting function. However, our analysis of C1A and R344A mutations argues that Asn1p filaments are unlikely to have acquired a second, non-enzymatic role in cellular function.

While our studies of the Asn1/2p system have not revealed a moonlighting function for Asn1p filaments, they have laid the groundwork for exploiting differences in the paralogs to identify mutations that selectively block filament formation. Our analysis of R344A mutation argues that the Asn1p synthetase domain plays a critical role in polymerization. Future work focused on testing the amino acid differences between the synthetase domains of Asn1p and Asn2p for their effects on polymerization is likely to identify the critical residues that either comprise or regulate the polymerization interface. The identification of the interface will provide critical insights for identifying asparagine synthetases in other organisms that are capable of polymerization as well as suggest novel ways to target human asparagine synthetase induced in the acute lymphoblastic leukemia patients with asparaginase resistance^19,20^.

## Methods

### Bacteria, yeast strain, growth and selection media

*Escherichia coli* DH5α was used for transformation and propagation of the recombinant plasmid. LB medium [0.5% (w/v) yeast extract (BD), 1% (w/v) Bacto-tryptone (BD), 1% (w/v) sodium chloride (BDH Prolabo)] supplemented with 100 μg/ml ampicillin (PanReac Applichem) was used for selection. Bacterial cultures were maintained at 37 °C.

Yeast BY4741 (*MATa his3Δ1 leu2Δ0 met15Δ0 ura3Δ0*), used as a background strain for yeast chromosomal gene modifications, yeast *ASN1::GFP*, used as a base strain for colocalization assay, and yeast *ASN2::GFP*, used as a base strain for assembly dependence assay, were purchased from Thermo Fisher Scientific. YPD [1% (w/v) yeast extract (BD), 2% (w/v) Bacto-peptone (BD), and 2% (w/v) dextrose (Sigma-Aldrich)] medium was used for general growth. G418 (PanReac Applichem) (400 μg/ml final concentration) and hygromycin B (Merck) (200 μg/ml final concentration) were used for selecting yeast transformants. All yeast strains were maintained at 30 °C.

### Yeast *ASN1::GFP ASN2::mCherry* construction for colocalization assay

Yeast *ASN1::GFP ASN2::mCherry* was created using yeast *ASN1::GFP* from the yeast GFP collection as a base strain. pBS34 was used as a DNA template for making DNA cassette harboring (sequence in order from 5′ to 3′): 50 nt upstream of the *ASN2* stop codon, *mCherry* coding sequence, kanamycin resistance gene, and 50 nt downstream of the *ASN2* stop codon. The PCR reaction was set up using the KOD Hot Start DNA Polymerase kit (Merck). Yeast *ASN1::GFP* was transformed with the purified DNA cassette using lithium acetate/polyethylene glycol transformation method. YPD medium supplemented with G418 was used for selection. PCR was employed to verify that *mCherry* was successfully fused to *ASN2* in the yeast genome. The primers (synthesized by ValueGene, USA) used for mCherry fusion are shown in Supplementary Table S1.

### Yeast *ASN1::GFP asn2Δ* and *ASN2::GFP asn1Δ* constructions for assembly dependence assay

Yeast *ASN1::GFP asn2Δ* was created using BY4741 as a background strain. First, pFA6a-GFP-kanMX6 was used as a DNA template for making DNA cassette harboring (sequence in order from 5′ to 3′): 50 nt upstream of the *ASN1* stop codon, *GFP* coding sequence, kanamycin resistance gene, and 50 nt downstream of the *ASN1* stop codon. The PCR reaction was set up using the KOD Hot Start DNA Polymerase kit. Yeast BY4741 was transformed with the purified DNA cassette using lithium acetate/polyethylene glycol transformation method. YPD medium supplemented with G418 was used for selection. PCR was employed to verify that *GFP* was successfully fused to *ASN1* in the yeast genome. Then, pFA6a-hphMX6 was used as a DNA template for making DNA cassette harboring (sequence in order from 5′ to 3′): 50 nt upstream of the *ASN2* start codon, hygromycin resistance gene, and 50 nt downstream of the *ASN2* stop codon. The PCR reaction was set up using the KOD Hot Start DNA Polymerase kit. Yeast *ASN1::GFP* was transformed with the purified DNA cassette using lithium acetate/polyethylene glycol transformation method. YPD medium supplemented with G418 and hygromycin B was used for selection. PCR was then used to verify that *ASN2* was successfully deleted from the yeast genome.

To create yeast *ASN2::GFP asn1Δ*, yeast *ASN2::GFP* from the yeast GFP collection was used as a base strain. pFA6a-hphMX6 was used as a DNA template for making DNA cassette harboring (sequence in order from 5′ to 3′): 50 nt upstream of the *ASN1* start codon, hygromycin resistance gene, and 50 nt downstream of the *ASN1* stop codon. The PCR reaction was set up using the KOD Hot Start DNA Polymerase kit. Yeast *ASN2::GFP* was transformed with the purified DNA cassette using lithium acetate/polyethylene glycol transformation method. YPD medium supplemented with hygromycin B was used for selection. PCR was then used to verify that *ASN1* was successfully deleted from the yeast genome.

The primers (synthesized by ValueGene, USA) used for GFP fusion and gene deletions are shown in Supplementary Table S1.

### Construction of recombinant plasmid

pFA6a-GFP-kanMX6 was used for molecular cloning of *ASN1*. The *ASN1* coding sequence was amplified by PCR from the isolated genomic DNA of yeast BY4741, using KOD Hot Start DNA Polymerase, and then directionally cloned into pFA6a-GFP-kanMX6 at *Sal*I and *Sma*I restriction recognition sites (the restriction endonucleases purchased from New England Biolabs). The resulting plasmid was named pFA6a-ASN1-GFP-kanMX6. The primers (synthesized by ValueGene, USA) used for PCR are shown in Supplementary Table S1.

### PCR-based site-directed mutagenesis

pFA6a-ASN1-GFP-kanMX6 was used as a DNA template to introduce C1A, R344A, and C1A-R334A mutations to the *ASN1* coding sequence within the plasmid. All primers used for mutagenization are listed in Supplementary Table S1. Primers were first phosphorylated at their 5′ ends with T4 Polynucleotide Kinase (New England Biolabs). PCR-based site-directed mutagenesis reactions were set up using KOD Hot Start DNA Polymerase. The plasmid template was removed by *Dpn*I treatment. The mutagenesis PCR products were purified using PureLink PCR Purification Kit (Thermo Fisher Scientific). Ligations were set up using T4 DNA Ligase (New England Biolabs) to make mutagenized PCR products (linear plasmids) become circular prior to bacterial transformation. After transformation using heat shock method, plasmids were isolated from the selected transformants using PureLink Quick Plasmid Miniprep Kit (Thermo Fisher Scientific), and were then verified by DNA sequencing (Retrogen, USA). The resulting plasmids after mutagenization were pFA6a-ASN1(C1A)-GFP-kanMX6, pFA6a-ASN1(R344A)-GFP-kanMX6, and pFA6a-ASN1(C1A-R344A)-GFP-kanMX6, respectively.

### Yeast chromosomal gene modifications to construct yeast *asn1(C1A or R344A or C1A-R344A)::GFP asn2Δ* for structure-function analysis

PCR-based engineering of yeast genome was employed for creating all yeast strains used in this study. The *asn2Δ* strain was constructed to be used as a background strain. Briefly, pFA6a-hphMX6 was used as a DNA template for making DNA cassette harboring (sequence in order from 5′ to 3′): 50 nt upstream of the *ASN2* start codon, hygromycin resistance gene, and 50 nt downstream of the *ASN2* stop codon. The PCR reaction was set up using the KOD Hot Start DNA Polymerase kit. Yeast BY4741 was transformed with the purified DNA cassette using lithium acetate/polyethylene glycol transformation method. YPD medium supplemented with hygromycin B was used for selection. PCR was then used to verify that *ASN2* was successfully deleted from the yeast genome.

After having yeast *asn2Δ* background strain, the recombinant plasmids pFA6a-ASN1(C1A)-GFP-kanMX6, pFA6a-ASN1(R344A)-GFP-kanMX6, and pFA6a-ASN1(C1A-R344A)-GFP-kanMX6 were used as DNA templates for making the DNA cassettes harboring (sequence in order from 5′ to 3′): 50 nt upstream of the *ASN1* start codon, *ASN1* coding sequence (with corresponding mutation), GFP, kanamycin resistance gene, and 50 nt downstream of the *ASN1* stop codon. The PCR reactions were set up using the KOD Hot Start DNA Polymerase kit. Yeast *asn2Δ* was transformed with the purified DNA cassettes using lithium acetate/polyethylene glycol transformation method. YPD medium supplemented with G418 was used for selection. The positive yeast transformants were initially screened under the fluorescence microscope, and were then confirmed by sending out the PCR products of the genomic DNA isolated from the selected yeast constructs to be verified for DNA sequencing (Retrogen, USA). The primers for making the DNA cassettes for yeast transformations and sequencing primers are shown in Supplementary Table S1.

### Cell counting, imaging, and length distribution analysis

Yeast samples were grown in YPD at 30 °C with shaking to the growth stages as indicated. Cells were fixed with formaldehyde by using 100 μl 37% formaldehyde per 1 ml of cell culture. After fixing at room temperature for 15 min with shaking in the dark, the cells were washed twice with sterile water, and then resuspended in either 1xPBS or 1 M sorbitol. Wet slides were prepared by dropping the fixed cell suspension (about 10 μl) onto a slide (Shandon Superfrost Plus, Thermo Scientific), covering with a coverslip (Menzel Gläser, Thermo Scientific), blotting off excess liquid to prevent cells from floating around, and then sealing the edges of the coverslip with nail polish.

For counting cells under fluorescence microscope, yeast cells were randomly counted in 5 different fields of view on the slide (about 250 cells in total). Then, the percentage of cells with Asn1p-GFP structures was calculated. The average ± SEM of three independent experiments was reported for each condition, and student’s t-test was used for statistical analyses (GraphPad Prism version 7.03).

Images were taken with the Carl Zeiss LSM800 with AiryScan using Plan-Apochromat 63×/1.4 Oil DIC ∞/0.17 objective lens with Zen Blue software version 2.1.57.1000 (the Advanced Cell Imaging Center, Institute of Molecular Biosciences, Mahidol University).

The length distribution analysis was performed after cell imaging and maximal projection processing. 300 structures of each strain were analyzed for maximum, minimum, and average length using Zen Blue software version 2.1.57.1000.

### Western blot analysis

SDS-PAGE and Western blotting were performed with a standard protocol. All indicated yeast strains were grown in YPD for 1 day and 5 days at 30 °C with shaking. Five OD_600_ cells (1-day cultures) or 10 OD_600_ cells (5-day cultures) were collected to prepare 200-µl protein samples (loading 20 µl/sample; 8% SDS-PAGE). Asn1p-GFP expression levels were detected using (1:500) rabbit anti-human asparagine synthetase (hASNS) antibody (CA5498, Covance; test bleed#2, purified) and (1:5,000) HRP conjugated donkey anti-rabbit IgG (GE Healthcare). Pgk1p expression levels were detected using (1:20,000) mouse anti-PGK1 monoclonal antibody (22C5D8, Thermo Fisher Scientific) and (1:2,500) HRP conjugated sheep anti-mouse IgG (GE Healthcare). Each experiment used the same blot for probing with anti-hASNS and anti-PGK1, one at a time. Stripping buffer [62.5 mM Tris-HCl pH 6.8, 2% (w/v) SDS, 0.7% (w/v) BME] was used to remove previous antibody from the blot prior to addition of the other antibody. Full blots are shown in Supplementary Fig. S5.

## Electronic supplementary material

Supplementary Information

## Data Availability

All data generated or analyzed during this study are available from the corresponding authors on reasonable request.
