# Supplementary material for: Saccharomyces cerevisiae ASN1 and ASN2 are asparagine synthetase paralogs that have diverged in their ability to polymerize in response to nutrient stress
Source: Sci Rep. 2019 Jan 22;9:278. doi: 10.1038/s41598-018-36719-z (PMC6342913; doi:10.1038/s41598-018-36719-z)
Supplement: Supplementary file 1 — Supplementary Information [file 41598_2018_36719_MOESM1_ESM.pdf]

Supplementary Information

Saccharomyces cerevisiae ASN1 and *ASN2* are asparagine synthetase paralogs that have diverged in their ability to polymerize in response to nutrient stress

Chalongrat Noree[1,*], Naraporn Sirinonthanawech[1], and James E. Wilhelm[2,*]

[1]Institute of Molecular Biosciences, Mahidol University, 25/25 Phuttamonthon 4 Road, Salaya, Phuttamonthon, Nakhon Pathom 73170 Thailand

[2]Section of Cell and Developmental Biology, Division of Biological Sciences, University of California, San Diego, 9500 Gilman Drive (MC 0347), La Jolla, CA 92093-0347 USA

***Correspondence:**
Chalongrat Noree
Tel: +66-2-4419003
Fax: +66-2-4411013
Email: chalongrat.nor@mahidol.edu

James E. Wilhelm
Tel: 1-858-534-9541
Fax: 1-858-534-7214
Email: jwilhelm@ucsd.edu

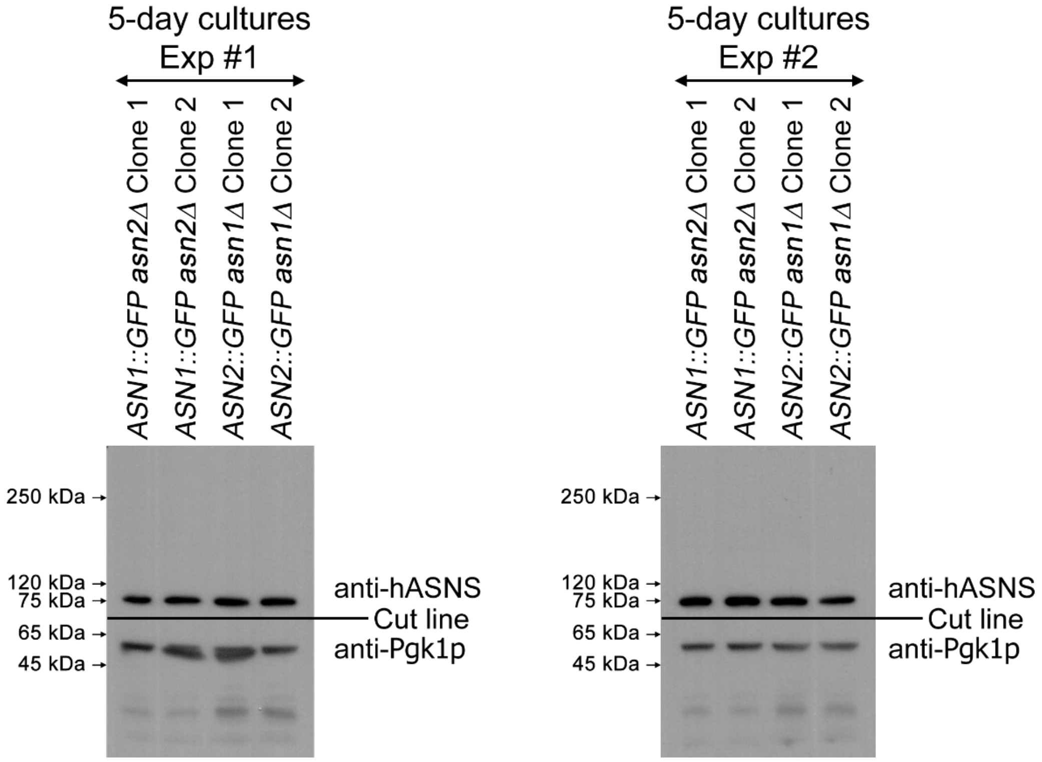

Supplementary Figure S1. Expression levels of Asn1p-GFP (in *asn2*Δ background) vs. Asn2p-GFP (in *asn1*Δ background). Two different clones of yeast *ASN1::GFP asn2Δ* and *ASN2::GFP asn1Δ* were grown in liquid YPD at 30°C for 5 days with shaking. Then, 10 OD_{600} cells were taken from each culture to prepare whole cell extract for SDS-PAGE and western blot analysis. Each membrane was cut into 2 pieces between 75 and 65 kDa of the pre-stained protein ladder. The upper cuts were used to detect GFP-tagged Asn1/2p-GFP with anti-hASNS (91.7 kDa and 91.9 kDa, respectively). The lower cuts were used to detect Pgk1p (as internal loading control) (44.7 kDa). Both were later assembled and developed the chemiluminescent signal together. Two independent experiments performed to confirm the results.

```
                10        20        30        40        50        60        70        80        90       100
E.coli  MCSIFGVFDIKTDAVELRKKALELSRLMRHRGPDWSGIYASDNAILAHERLSIVDVNAGAQPLYNQQK-THVLAVNGEIYNHQALRAEYGDRYQFQTGSD
Yeast   MCGIFAAFR-HEDVHRYKPKALQLSKRIRHRGPDWSGNAIKNSTIFVHERLAIVGVESGAQPITSSDG-EYMLCVNGEIYNHIQLREECAD-YEFGTLSD
Human   MCGIWALFGSDDCLSVQCLSAMKIAHRGPD-AFRFENVNGYTNCCFGFHRLAVVDPLFGMQPIRVKKYPYLWLCYNGEIYNHKKMQQHFEF--EYQTKVD

               110       120       130       140       150       160       170       180       190       200
E.coli  CEVILALYQEKGPE-FLDDLQGMFAFALYDSEKDAYLIGRDHLGIIPLYMGYDEHG--QLYVASEMKALVPVCRTIKEFPAGSYLWS------------
Yeast   CEPIIPMYLKHDID-APKYLDGMFAWTLYDAKQDRIVAARDPIGITTLYMGRSSASPKTVYFASELKCLTDDCDTITAFPPGHVYDS------------
Human   GEIILHLYDKGGIEQTICMLDGVFAFVLLDTANKKVFLGRDTYGVRPLFKAMTEDG--FLAVCSEAKGLVTLKHSATPFLKVEPFLPGHYEVLDLKPNGK

               210       220       230       240       250       260       270       280       290       300
E.coli  -QDGEIRSYYHRDWFDYDAVKDNVTDKNE----------LRQALEDSVKSHLMSDVPYGVLLSGGLDSSIISAITKKYAARRVEDQERS-----------
Yeast   -KTDKITRYFTPDWLDEKRIPSTPIDYMA----------IRHSLEKAVRKRLMAEVPYGVLLSGGLDSSLIASIAARETAKATNDVEPSTYDSKARHLAG
Human   VASVEMVKYHHCRDVPLHALYDNVEKLFPGFEIETVKNNLRILFNNAVKKRLMTDRRIGCLLSGGLDSSLVAATLLKQLKEAQVQ--------------

               310       320       330       340       350       360       370       380       390       400
E.coli  --------EAWWPQLHSFAVGLPGSPDLKAAQEVANHLGTVHHEIHFTVQEGLDAIRDVIYHIETYDVTTIRASTPMYLMSRKIKAMG-IKMVLSGEGSD
Yeast   IDDDGKLHTAGWTSLHSFAIGLPNAPDLQAARKVAKFIGSIHHEHTFTLQEGLDALDDVIYHLETYDVTTIRASTPMFLLSRKIKAQG-VKMVLSGEGSD
Human   ------------YPLQTFAIGMEDSPDLLAARKVADHIGSEHYEVLFNSEEGIQALDEVIFSLETYDITTVRASVGMYLISKYIRKNTDSVVIFSGEGSD
                                                                              ▲

               410       420       430       440       450       460       470       480       490       500
E.coli  EVFGGYLYFHKAPNAKELHEETVRKLLALHMYDCARANKAMSAWGVEARVPFLDKKFLDVAMRINPQDKMCG--NGKMEKHILRECFEA--------YLP
Yeast   EIFGGYLYFAQAPSAAEFHTESVQRVKNLHLADCLRANKSTMAWGLEARVPFLDREFLQLCMNIDPNEKMIKPKEGRIEKYILRKAFDTTGEPDAKPYLP
Human   ELTQGYIYFHKAPSPEKAEEESERLLRELYLFDVLRADRTTAAHGLELRVPFLDHRFSSYYLSLPPEMRIPK---NGIEKHLLRETFEDSN------LIP

               510       520       530       540       550       560       570       580       590       600
E.coli  ASVAWRQKEQFSDGVG---YSWIDTLKEVAAQQVSDQQLETARFRFPYNTPTSKEAYLYREIFEELFPLPSAAECVPGGPSVACSSAKAIEWDEAFKKMD
Yeast   EEILWRQKEQFSDGVG---YSWIDGLKDTAEAVISDEMFASPKAEWGSDIPTTKEAFWYRLKFDALFPQKTVADTVMR-------WIPKADWGCA----E
Human   KEILWRPKEAFSDGITSVKNSWFKILQEYVEHQVDDAMMANAAQKFPFNTPKTKEGYYYRQVFERHYPGR-------------ADWLSHYWMPKWINAT

               610
E.coli  DPSGRAVGVHQSAYK-
Yeast   DPSGRYAQIHEKHIE-
Human   DPSARTLTHYKSAVKA
```

Supplementary Figure S2. Amino acid sequence alignment of *E. coli*, yeast, and human asparagine synthetases. Amino acid sequences of *E. coli* K12 asparagine synthetase B (P22106), yeast Asn1p (YPR145W, SGDID:S000006349), and human ASNS (P08243) were aligned. Yeast Asn1p shows: 37% identity and 53% similarity with human ASNS, and 48% identity and 64% similarity with *E. coli* asparagine synthetase B, respectively. The multiple alignment was performed with BioEdit version 7.1.9. The red arrow indicates arginine residue at position 325 of *E. coli*, 344 of yeast, and 340 of human asparagine synthetase, respectively.

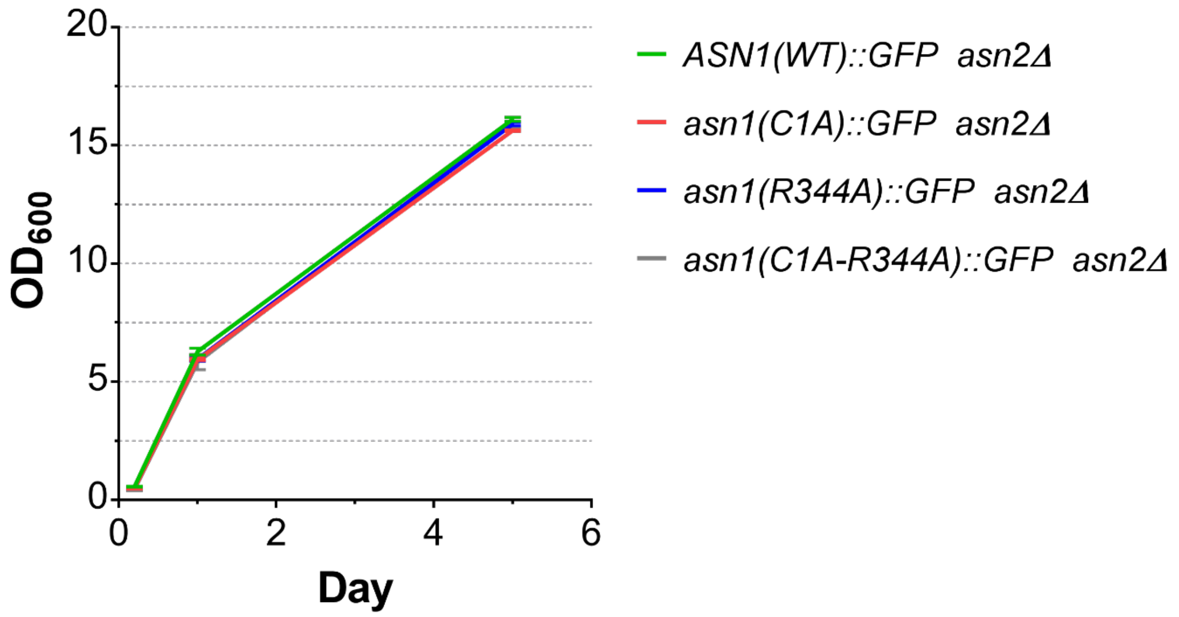

Yeast strain	Clone #	OD_{600} (Average ± SEM)		
		Log-phase	Day 1	Day 5
*ASN1(**WT**)::GFP asn2Δ*	1	0.57 ± 0.03	6.29 ± 0.10	16.44 ± 0.27
	2	0.57 ± 0.02	6.27 ± 0.14	16.11 ± 0.09
*asn1(**C1A**)::GFP asn2Δ*	1	0.47 ± 0.02	5.94 ± 0.05	15.63 ± 0.04
	2	0.46 ± 0.03	5.90 ± 0.04	15.60 ± 0.12
*asn1(**R344A**)::GFP asn2Δ*	1	0.49 ± 0.03	5.96 ± 0.10	15.90 ± 0.06
	2	0.54 ± 0.02	6.23 ± 0.20	16.11 ± 0.22
*asn1(**C1A-R344A**)::GFP asn2Δ*	1	0.46 ± 0.07	5.84 ± 0.33	15.99 ± 0.21
	2	0.43 ± 0.07	5.97 ± 0.05	16.05 ± 0.13

Supplementary Figure S3. Growth curve comparison of yeast *ASN1(WT)::GFP asn2Δ*, *asn1(C1A)::GFP asn2Δ*, *asn1(R344A)::GFP asn2Δ*, and *asn1(C1A-R344A)::GFP asn2Δ*. These yeast strains were cultured in YPD at 30°C, and measured their optical density (OD_{600}) at exponential phase of growth, day 1, and day 5, respectively. Three independent experiments were performed and reported as average ± SEM. Clone#1 of each strain was used to make a growth curve. Summary table is shown below the growth curve.

```
               10        20        30        40        50        60        70        80        90       100
       ....|....|....|....|....|....|....|....|....|....|....|....|....|....|....|....|....|....|....|....|
Asn1p  MCGIFAAFRHEDVHRYKPKALQLSKRIRHRGPDWSGNAIKNSTIFVHERLAIVGVESGAQPITSSDGEYMLCVNGEIYNHIQLREECADYEFGTLSDCEP
Asn2p  MCGIFAAFKHEDIHNFKPKALQLSKKIRHRGPDWSGNAVMNSTIFVHERLAIVGLDSGAQPITSADGEYMLGVNGEIYNHIQLREMCSDYKFQTFSDCEP

              110       120       130       140       150       160       170       180       190       200
       ....|....|....|....|....|....|....|....|....|....|....|....|....|....|....|....|....|....|....|
Asn1p  IIPMYLKHDIDAPKYLDGMFAWTLYDAKQDRIVAARDPIGITTLYMGRSSASPKTVYFASELKCLTDDCDTITAFPPGHVYDSKTDKITRYFTPDWLDEK
Asn2p  IIPLYLEHDIDAPKYLDGMFAFCLYDSKKDRIVAARDPIGVVTLYMGRSSQSPETVYFASELKCLTDVCDSIISFPPGHVYDSETDKITRYFTPDWLDEK

              210       220       230       240       250       260       270       280       290       300
       ....|....|....|....|....|....|....|....|....|....|....|....|....|....|....|....|....|....|....|
Asn1p  RIPSTPIDYMAIRHSLEKAVRKRLMAEVPYGVLLSGGLDSSLIASIAARETAKATNDV-EPSTYDSKARHLAGIDDDGKLHTAGWTSLHSFAIGLPNAPD
Asn2p  RIPSTPVDYHAIRHSLEKAVRKRLMAEVPYGVLLSGGLDSSLIAAIAARETEKANADANEDNNVDEK--QLAGIDDQGHLHTSGWSRLHSFAIGLPNAPD

              310       320       330       340       350       360       370       380       390       400
       ....|....|....|....|....|....|....|....|....|....|....|....|....|....|....|....|....|....|....|
Asn1p  LQAARKVAKFIGSIHHEHTFTLQEGLDALDDVIYHLETYDVTTIRASTPMFLLSRKIKAQGVKMVLSGEGSDEIFGGYLYFAQAPSAAEFHTESVQRVKN
Asn2p  LQAARKVAKFIGSIHHEHTFTLQEGLDALDDVIYHLETYDVTTIRASTPMFLLSRKIKAQGVKMVLSGEGSDEIFGGYLYFAQAPSAAEFHTESVQRVKN

              410       420       430       440       450       460       470       480       490       500
       ....|....|....|....|....|....|....|....|....|....|....|....|....|....|....|....|....|....|....|
Asn1p  LHLADCLRANKSTMAWGLEARVPFLDREFLQLCMNIDPNEKMIKPKEGRIEKYILRKAFDTTGEPDAKPYLPEEILWRQKEQFSDGVGYSWIDGLKDTAE
Asn2p  LHLADCLRANKSTMAWGLEARVPFLDKDFLQLCMNIDPNEKMIKPKEGRIEKYILRKAFDTTDEPDVKPYLPEEILWRQKEQFSDGVGYSWIDGLRDTAE

              510       520       530       540       550       560       570
       ....|....|....|....|....|....|....|....|....|....|....|....|....|....|....|
Asn1p  AVISDEMFASPKAEWGSDIPTTKEAFWYRLKFDALFPQKTVADTVMRWIPKADWGCAEDPSGRYAQIHEKHIE-*
Asn2p  RAISDAMFANPKADWGDDIPTTKEAYWYRLKFDAWFPQKTAADTVMRWIPKADWGCAEDPSGRYAKIHEKHVSA*
```

Supplementary Figure S4. Pairwise amino acid sequence alignment of yeast asparagine synthetase paralogs. The alignment of Asn1p (YPR145W, SGDID:S000006349) and Asn2p (YGR124W, SGDID:S000003356) shows 88% identity and 94% similarity of their amino acid sequences. The pairwise alignment (optimal GLOBAL) was performed with BioEdit version 7.1.9.

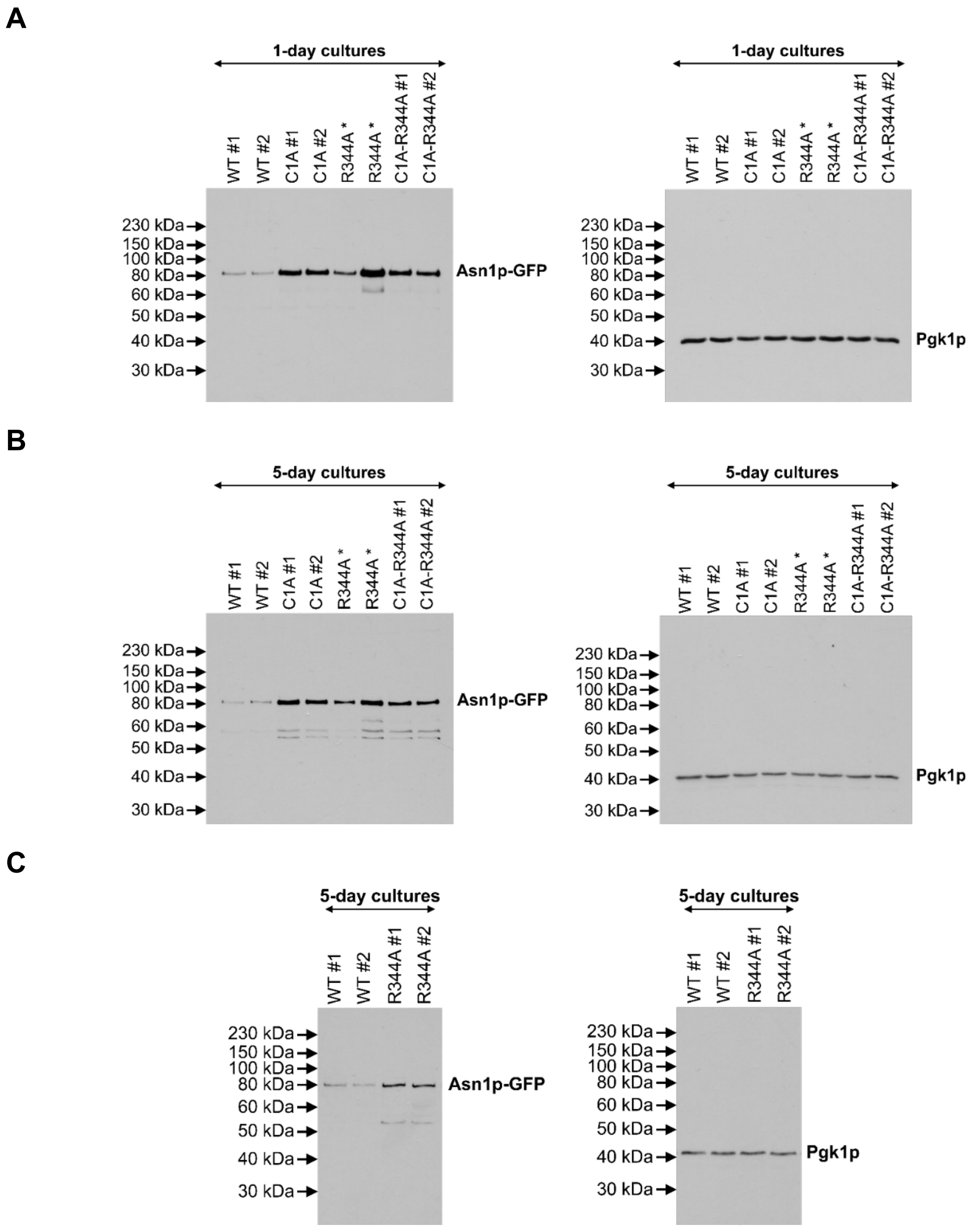

Supplementary Figure S5. Defective structure formation of R344A and C1A-R344A Asn1p-GFP was not caused by their protein expression levels. Yeast *ASN1(WT)::GFP asn2Δ*, *asn1(C1A)::GFP asn2Δ*, *asn1(R344A)::GFP asn2Δ*, and *asn1(C1A-R344A)::GFP asn2Δ* were grown in YPD for 1 day (**A**) and 5 days (**B-C**) at 30°C with shaking. Five OD_{600} cells (1-day cultures) or 10 OD_{600} cells (5-day cultures) were collected for preparing 200-μl whole cell extracts. Twenty μl of each protein sample were resolved on 8% acrylamide gel. Anti-hASNS antibody was used to detect Asn1p-GFP (91.75 kDa). Anti-PGK1 monoclonal antibody was used to detect yeast 3-phosphoglycerate kinase (44.74 kDa) as an internal loading control. Each experiment used the same blot for probing with anti-hASNS and anti-PGK1, one at a time. Stripping buffer [62.5 mM Tris-HCl pH 6.8, 2% (w/v) SDS, 0.7% (w/v) BME] was used

to remove previous antibody from the blot prior to addition of the other antibody. The clones of *asn1(R344A)::GFP asn2Δ,* marked with asterisk in (**A**) and (**B**), were not used in this study as they showed unequal expression levels (although they both showed the Asn1p-GFP polymerization defect). The clones of *asn1(R344A)::GFP asn2Δ* used for analysis in this study were shown in (**C**).

Supplementary Table S1. List of primers used for recombinant DNA cloning, making DNA cassettes for yeast transformation, verifying yeast transformants, and DNA sequencing.

Primer Code	Sequence (5’ to 3’)	Description	Used with	PCR Product Size
**For creating yeast *ASN1::GFP ASN2::mCherry* (colocalization assay)**<br>**DNA cassette carrying (5’ to 3’): 50 nt upstream of the *ASN2* stop codon, *mCherry* coding sequence, kanamycin resistance gene, and 50 nt downstream of the *ASN2* stop codon**<br>**DNA template: pBS34**				
CN0033	5’-AAGATCCTTCAGGTAGATACGCCAAAATACACGAAAAGCACGTCAGTGCTGGTCGACGGATCCCCGGG -3’	Forward, with 50 nt upstream of ***ASN2*** stop codon, sequence homology to pBS34 (underlined)	JW1995	2,533 bp
JW1995	5’-CCGCATTTCTTGGTTCACTCGTCAATTATAAGAATACGATTGCGCTCGTAATCGATGAATTCGAGCTCG -3’	Reverse, with 50 nt downstream of ***ASN2*** stop codon, sequence homology to pBS34 (underlined)	CN0033	
**For verifying the success of introducing mCherry to *ASN2* in yeast genome (colocalization assay)**<br>**DNA template: genomic DNA isolated from selected yeast transformants**				
JW2041	5’- TTGATCCAAATGAAAAGATGATCAAG -3’	Forward, located at nt1301-1326 of ***ASN2*** coding sequence	JW1775	1,154 bp
JW1775	5'- CTACTTGTACAGCTCGTCCATGCC -3'	Reverse, located at nt688-711 of **mCherry** coding sequence	JW2041	
**For creating yeast *asn1Δ* (assembly dependence assay)**<br>**DNA cassette carrying (5’ to 3’): 50 nt upstream of the *ASN1* start codon, hygromycin resistance gene, and 50 nt downstream of the *ASN1* stop codon**<br>**DNA template: pFA6a-hphMX6**				
JW2201	5’-AAAAGTATAACTTGCTTTACGCTAAGGATATAAATCGGACGTAACTTAAGGGTCGACGGATCCCCGGG - 3'	Forward, with 50 nt upstream of ***ASN1*** start codon, sequence homology to pFA6a-hphMX6 (underlined)	JW2202	1,811 bp
JW2202	5’-AAATATCTATAAGATTAATCCATAATTCTTTTTCTATTTTTTAATGTTATATCGATGAATTCGAGCTCG -3’	Reverse, with 50 nt downstream of ***ASN1*** stop codon, sequence homology to pFA6a-hphMX6 (underlined)	JW2201	
**For creating yeast *asn2Δ* (assembly dependence assay)**<br>**DNA cassette carrying (5’ to 3’): 50 nt upstream of the *ASN2* start codon, hygromycin resistance gene, and 50 nt downstream of the *ASN2* stop codon**<br>**DNA template: pFA6a-hphMX6**				
JW2193	5’-TTGCTTCCCAGTTAAGCTCACCAAAACACAAATACTCACTACAATACAATGGTCGACGGATCCCCGGG -3’	Forward, with 50 nt upstream of ***ASN2*** start codon, sequence homology to pFA6a-hphMX6 (underlined)	JW2194	1,811 bp
JW2194	5’-CCGCATTTCTTGGTTCACTCGTCAATTATAAGAATACGATTGCGCTCGTAATCGATGAATTCGAGCTCG -3’	Reverse, with 50 nt downstream of ***ASN2*** stop codon, sequence homology to pFA6a-hphMX6 (underlined)	JW2193	
**For verifying the success of deleting *ASN1* and *ASN2* from yeast genome (assembly dependence assay)**<br>**DNA template: genomic DNA isolated from selected yeast transformants**				
JW2010	5’- CTGCCCACTCGAGATGACAAATA -3’	Forward, located 200 nt upstream of ***ASN1*** start codon	JW1986	1,355 bp
JW1986	5’- CTCCATACAAGCCAACCACG -3’	Reverse, located at nt713-732 of hygromycin	JW2010	

Primer Code	Sequence (5’ to 3’)	Description	Used with	PCR Product Size
		resistance coding sequence		
JW2009	5’- CATTGACTCATGGCAAGATTTCTCC -3’	Forward, located 200 nt upstream of ***ASN2*** start codon	JW1986	1,355 bp
JW1986	5’- CTCCATACAAGCCAACCACG -3’	Reverse, located at nt713-732 of hygromycin resistance coding sequence	JW2009	
**For cloning *ASN1* into pFA6a-GFP-kanMX6**<br>**DNA template: genomic DNA isolated from yeast BY4741**				
JW2022	5’- CTTGTCGACATGTGTGGTATTTTCGCCGCTTTC -3’	Forward, for cloning ***ASN1***, ***Sal*I** site underlined	JW2023	1,734 bp
JW2023	5’- CTTCCCGGGTTCGATATGTTTTTCATGAATTTGGGCATATC -3’	Reverse, for cloning ***ASN1***, ***Sma*I** site underlined	JW2022	
**For site-directed mutagenesis of pFA6a-ASN1-GFP-kanMX6 (to introduce C1A or R344A mutation into *ASN1* coding sequence)**<br>**DNA template: pFA6a-ASN1-GFP-kanMX6**				
JW2259	5’- GACGTTACCACTATC**GCA**GCTTCCACTCCAATG -3’	Forward, with **R344A** mutation (in bold)	JW2260	6,593 bp
JW2260	5’- GTAAGTTTCCAAATGGTAGATCAC - 3'	Reverse	JW2259	
JW2261	5’- CTGCAGGTCGACATG**GCT**GGTATTTTCGCCGCT -3’	Forward, with **C1A** mutation (in bold)	JW2262	6,593 bp
JW2262	5’- CGTACGAAGCTTCAGCTGG -3’	Reverse	JW2261	
**For making yeast *ASN1(R344A)::GFP***<br>**DNA cassette carrying (5’ to 3’): *ASN1* coding sequence (with R344A mutation), kanamycin resistance gene, and 50 nt downstream of the *ASN1* stop codon**<br>**DNA template: pFA6a-ASN1(R344A)-GFP-kanMX6**				
JW2038	5’- CGCATTCCTTCCACCCCAATAG -3’	Forward, located at nt601-622 of ***ASN1*** coding sequence	JW2160	3,590 bp
JW2160	5’- AAATATCTATAAGATTAATCCATAATTCTTTTTCTATTTTTTAATGTTATATCGATGAATTCGAGCTCG -3’	Reverse, with 50 nt downstream of ***ASN1*** stop codon, sequence homology to pFA6a-ASN1-GFP-kanMX6 (underlined)	JW2038	
**For making yeast *ASN1(C1A)::GFP* and yeast *ASN1(C1A-R344A)::GFP***<br>**DNA cassette carrying (5’ to 3’): 50 nt upstream of the *ASN1* start codon, *ASN1* coding sequence (with desired mutation), kanamycin resistance gene, and 50 nt downstream of the *ASN1* stop codon**<br>**DNA template: pFA6a-ASN1(C1A)-GFP-kanMX6 or pFA6a-ASN1(C1A-R344A)-GFP-kanMX6**				
JW2276	5’- AAAAGTATAACTTGCTTTACGCTAAGGATATAAATCGGACGTAACTTAAG*ATG***GCT**GGTATTTTCGCCGCT -3’	Forward, with 50 nt upstream of ***ASN1*** start codon, start codon (italicized), **C1A** mutation (in bold)	JW2160	4,240 bp
JW2160	5’- AAATATCTATAAGATTAATCCATAATTCTTTTTCTATTTTTTAATGTTATATCGATGAATTCGAGCTCG -3’	Reverse, with 50 nt downstream of ***ASN1*** stop codon, sequence homology to pFA6a-ASN1-GFP-kanMX6 (underlined)	JW2276	
**For preparing PCR products to send out for DNA sequencing**<br>**DNA template: genomic DNA isolated from yeast *ASN1(C1A)::GFP*, yeast *ASN1(R344A)::GFP*, and yeast *ASN1(C1A-R344A)::GFP***				
JW2010	5’- CTGCCCACTCGAGATGACAAATA -3’	Forward, located 200 nt upstream of ***ASN1*** start codon	JW1623	2,829 bp

Primer Code	Sequence (5’ to 3’)	Description	Used with	PCR Product Size
JW1623	5’- GCGACCTCATACTATACCTG -3'	Reverse, located 184 nt downstream of ***GFP*** coding sequence	JW2010	
**For DNA sequencing**				
JW2010	5’- CTGCCCACTCGAGATGACAAATA -3’	Forward, located 200 nt upstream of ***ASN1*** start codon		
JW2038	5’- CGCATTCCTTCCACCCCAATAG -3’	Forward, located at nt601-622 of ***ASN1*** coding sequence		
JW2039	5’- ACATCGATCCAAATGAAAAGATG -3’	Forward, located at nt1301-1323 of ***ASN1*** coding sequence		
JW1623	5’- GCGACCTCATACTATACCTG -3'	Reverse, located 184 nt downstream of ***GFP*** coding sequence (in pFA6a-ASN1-GFP-kanMX6)		
